# Crystal structure of (1*RS*,21*SR*,22*RS*,24*SR*)-28-oxo-24-propyl-8,11,14-trioxa-24,27-di­aza­penta­cyclo[19.5.1.1^22,26^.0^2,7^.0^15,20^]octa­cosa-2,4,6,15(20),16,18-hexa­ene acetic acid monosolvate

**DOI:** 10.1107/S2056989016007556

**Published:** 2016-05-20

**Authors:** Truong Hong Hieu, Le Tuan Anh, Anatoly T. Soldatenkov, Nguyen Van Tuyen, Victor N. Khrustalev

**Affiliations:** a Institute of Chemistry, Vietnam Academy of Science and Technology, 18 Hoang Quoc Viet, Hanoi, Vietnam; b Department of Chemistry, Vietnam National University, 144 Xuan Thuy, Cau Giay, Hanoi, Vietnam; c Organic Chemistry Department, Peoples’ Friendship University of Russia, 6 Miklukho-Maklay St., Moscow 117198, Russian Federation; d Inorganic Chemistry Department, Peoples’ Friendship University of Russia, 6 Miklukho-Maklay St, Moscow, 117198, Russian Federation; e X-Ray Structural Centre, A. N. Nesmeyanov Institute of Organoelement Compounds, Russian Academy of Sciences, 28 Vavilov St, B-334, Moscow 119991, Russian Federation

**Keywords:** crystal structure, Petrenko–Kritchenko condensation, aza-14-crown-3-ether

## Abstract

The title compound, C_26_H_32_N_2_O_4_(*M*)·C_2_H_4_O_2_, (I), is the product of the Petrenko–Kritchenko condensation of *N*-propyl­piperidinone with 1,5-bis­(2-formyl­phen­oxy)-3-oxa­pentane and ammonium acetate. In *M*, the aza-14-crown-3-ether ring adopts a bowl conformation, with the configuration of the C—O—C—C —O—C—C—O—C polyether chain being *t*–*g* ^(−)^–*t*–*t*–*g* ^(+)^–*t* (*t* = *trans*, 180°; *g* = *gauche*, ±60°). The dihedral angle between the planes of the benzene rings fused to the aza-14-crown-4-ether moiety is 62.75 (5)°. The central piperidinone ring has a boat conformation, whereas the terminal piperidinone ring adopts a chair conformation. The boat conformation of the central piperidinone ring is supported by the bifurcated intra­molecular N—H⋯O hydrogen bond. In the crystal, each solvent mol­ecule is linked to mol­ecule *M via* strong O—H⋯N hydrogen bonding, forming hydrogen-bonded pairs of mol­ecules, which further inter­act through weak C—H⋯O hydrogen bonds, forming layers parallel to the *ac* plane.

## Abstract 2

The crystal structure of a product of the Petrenko–Kritchenko condensation of *N*-propyl­piperidone with 1,5-bis­(2-formyl­phen­oxy)-3-oxa­pentane and ammonium acetate was studied by X-ray diffraction

## Chemical context   

The design, synthesis and applications of macrocyclic ligands for coordination and supra­molecular chemistry have attracted very great attention from investigators over the last several decades (Hiraoka, 1978 ▸; Pedersen, 1988 ▸; Schwan & Warkentin, 1988 ▸; Gokel & Murillo, 1996 ▸; Bradshaw & Izatt, 1997 ▸). Recently, we have developed effective methods of synthesis of aza­crown ethers containing piperidine (Levov *et al.*, 2006 ▸, 2008 ▸; Anh *et al.*, 2008 ▸, 2012*a* ▸,*b* ▸,*c* ▸; Hieu *et al.* (2012*a* ▸,*b* ▸, 2013 ▸), perhydro­pyrimidine (Hieu *et al.*, 2011 ▸), perhydro­triazine (Khieu *et al.*, 2011 ▸) and bis­pidine (Komarova *et al.*, 2008 ▸; Sokol *et al.*, 2011 ▸) subunits.

**Figure 1:**
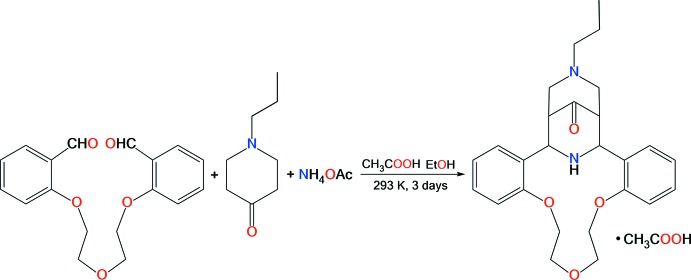
Petrenko–Kritchenko condensation of *N*-propyl­piperidinone with 1,5-bis­(2-formyl­phen­oxy)-3-oxa­pentane and ammonium acetate.

In attempts to apply this chemistry to obtain a macrocyclic ligand containing the *N*-propyl­substituted bis­pidine moiety, we studied the Petrenko–Kritchenko condensation of *N*-propyl­piperidinone with 1,5-bis­(2-formyl­phen­oxy)-3-oxa­pentane and ammonium acetate. The reaction proceeded smoothly to give the expected aza­crown system with a high yield of 73% (Fig. 1 ▸).

**Figure 2:**
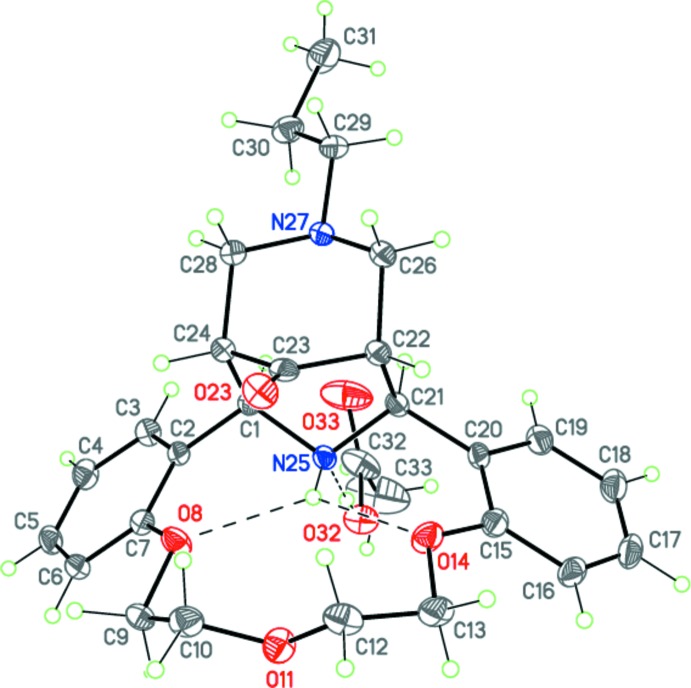
The mol­ecular structure of (I). Displacement ellipsoids are shown at the 50% probability level. H atoms are presented as small spheres of arbitrary radius. Dashed lines indicate the intra­molecular N—H⋯O and inter­molecular O—H⋯N hydrogen bonds.

The prepared compound was studied by X-ray diffraction analysis. It is a stable complex and crystallized as an acetic acid monosolvate, C_26_H_32_N_2_O_4_(*M*)·C_2_H_4_O_2_, (I) (Fig. 2 ▸). This finding seems to show the possibility of forming the second piperidine ring by the direct participation of the ammonium ion without the loss of its counter-ionic nature.
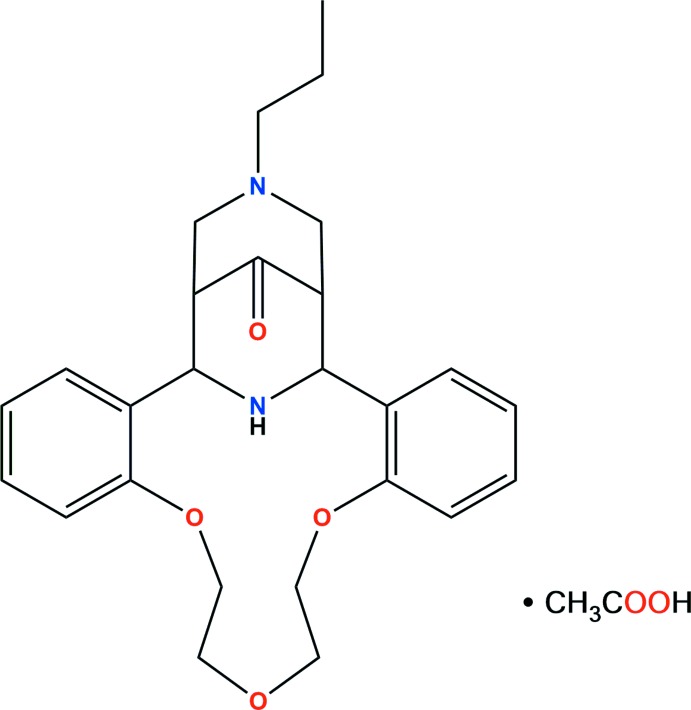


## Structural commentary   

The mol­ecule of *M* forms a robust hydrogen-bonded complex with an acetic acid mol­ecule by a strong inter­molecular O—H⋯N hydrogen bond (Fig. 2 ▸ and Table 1 ▸). The mol­ecule of *M* comprises a fused penta­cyclic system containing the aza-14-crown-3-ether macrocycle, two piperidinone and two benzene rings (Fig. 2 ▸). The aza-14-crown-3-ether ring adopts a bowl conformation. The conformation of the C7—O8—C9—C10 —O11—C12—C13—O14—C15 polyether chain is *t*–*g* ^(−)^–*t*–*t*–*g* ^(+)^–*t* (*t* = *trans*, 180°; *g* = *gauche*, ±60°). The dihedral angle between the planes of the benzene rings fused to the aza-14-crown-4-ether moiety is 62.75 (5)°. The central piperidinone ring has a boat conformation, whereas the terminal piperidinone ring adopts a chair conformation. Apparently, the conformation of the central piperidinone ring is determined by the bifurcated intra­molecular N—H⋯O hydrogen bond (Fig. 2 ▸ and Table 1 ▸). Both nitro­gen atoms N25 and N27 have a trigonal–pyramidal geometry (the sums of the bond angles are 326.9 and 335.2°, respectively). The bulk propyl substituent at the nitro­gen atom N27 occupies the more favourable equatorial position.

The mol­ecule of *M* possesses four asymmetric centers at the C1, C21, C22 and C24 carbon atoms and can have potentially numerous diastereomers. The crystal of (I) is racemic and consists of enanti­omeric pairs of *M* with the following relative configuration of the centers: *rac*-1R*, 21S*,22R*,24S*.

## Supra­molecular features   

**Figure 3:**
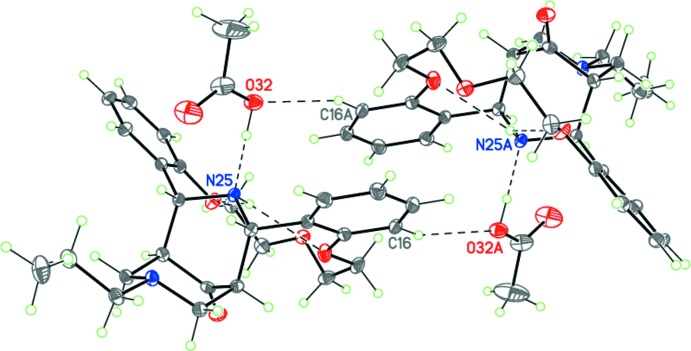
The centrosymmetric hydrogen-bonded dimer of (I). Dashed lines indicate the intra­molecular N—H⋯O and inter­molecular O—H⋯N and C—H⋯O hydrogen bonds [symmetry code: (A) −*x* + 2, −*y* + 1, −*z*].

**Figure 4:**
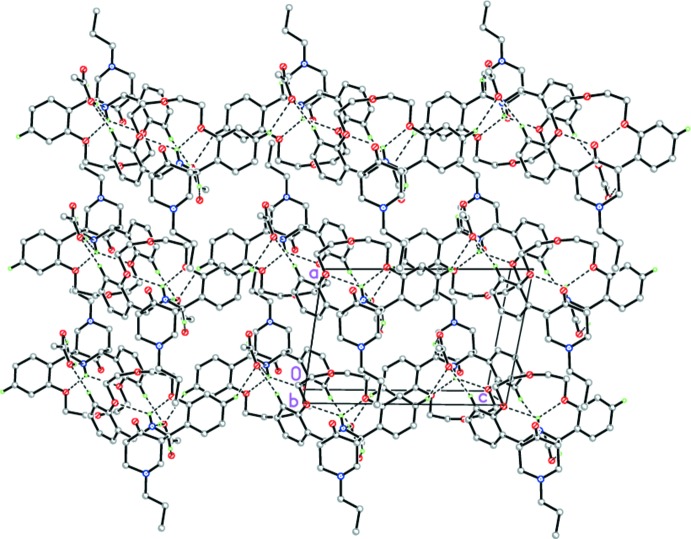
Crystal packing of (I) showing the layers parallel to the *ac* plane. Dashed lines indicate the intra­molecular N—H⋯O and inter­molecular O—H⋯N and C—H⋯O hydrogen bonds.

In the crystal, the hydrogen-bonded complex (I) forms centrosymmetric dimers by C—H⋯O hydrogen bonds (Fig. 3 ▸ and Table 1 ▸). The dimers inter­act through weak C—H⋯O hydrogen bonds, forming layers parallel to *ac* plane (Fig. 4 ▸ and Table 1 ▸).

## Synthesis and crystallization   

1,5-Bis(2-formyl-phen­oxy)-3-oxa­pentane was synthesized according to the procedure described previously (Levov *et al.*, 2008 ▸) and purified by recrystallization in ethanol.

Ammonium acetate (3.0 g, 39 mmol) was added to a solution of 1,5-bis­(2-formyl- phen­oxy)-3-oxa­pentane (3.14 g, 10.0 mmol) and *N*-propyl­piperidone (1.41 g, 10.0 mmol) in ethanol (30 mL) mixed with acetic acid (1 mL). The reaction mixture was stirred at 293 K for 3 d (monitoring by TLC until disappearance of the starting heterocyclic ketone spot). At the end of the reaction, the formed precipitate was filtered off, washed with ethanol and recrystallized from ethanol to give 3.60 g of colourless block-like crystals of (I) (yield 73%; m.p. = 490–492 K).

IR (KBr), ν/cm^−1^: 1602, 1728, 3263, 3463. ^1^H NMR (CDCl_3_, 400 MHz, 300 K): δ = 1.08 (*t*, 3H, CH_3_, *J* = 6.7), 1.25 (*m*, 2H, CH_2_ *CH_2_*CH_3_), 1.61 (*m*, 2H, N*CH_2_*CH_2_), 1.83 (*s*, 3H, *s*, 3H, CH_3_COO^−^), 2.49 (*m*, 4H, 2H23 and 2H25), 2.76 (*m*, 2H, H22 and H26), 3.12 (*br m*, 1H, NH), 3.86–4.10 (*m*, 8H, OCH_2_CH_2_OCH_2_CH_2_O), 4.83 (*m*, 2H, H1 and H21), 6.78–6.86 (*m*, 4H, H_arom_), 7.25–7.41 (*m*, 4H, H_arom_). ^13^C NMR (CDCl_3_, 80 MHz, 300 K): δ = 12.3 (CH_3_), 21.2 (CH_2_), 22.6 (CH_2_), 54.4 (CH_2_), 57.7 (CH_2_), 60.5 (CH_2_), 64.3 (CH_2_), 67.0 (CH), 79.1 (CH), 111.5 (C_arom_), 121.1 (C_arom_), 129.1 (C_arom_), 131.8 (C_arom_), 175.7 C=O). Analysis calculated for C_28_H_36_N_2_O_6_: C, 67.72; H, 7.31; N, 5.64. Found: C, 67.54; H, 7.42; N, 5.41.

## Refinement details   

Crystal data, data collection and structure refinement details are summarized in Table 2 ▸. The hydrogen atoms of the amino and hy­droxy groups were localized in the difference-Fourier maps and included in the refinement with fixed positional (using a riding model) and isotropic displacement parameters [*U*_iso_(H) = 1.2*U*_eq_(N) and 1.5*U*_eq_(O)]. The other hydrogen atoms were placed in calculated positions with C—H = 0.95–1.00 Å and refined in the riding model with fixed isotropic displacement parameters [*U*_iso_(H) = 1.5*U*_eq_(C) for the methyl group and 1.2*U*_eq_(C) for the other groups].

## Supplementary Material

Crystal structure: contains datablock(s) global, I. DOI: 10.1107/S2056989016007556/cv5505sup1.cif

Structure factors: contains datablock(s) I. DOI: 10.1107/S2056989016007556/cv5505Isup2.hkl

Supporting information file. DOI: 10.1107/S2056989016007556/cv5505Isup3.cml

CCDC reference: 1478354

Additional supporting information:  crystallographic information; 3D view; checkCIF report

## Figures and Tables

**Table 1:** Hydrogen-bond geometry (Å, °)

*D*—H⋯*A*	*D*—H	H⋯*A*	*D*⋯*A*	*D*—H⋯*A*
N25—H25⋯O8	0.91	2.27	2.867 (2)	123
N25—H25⋯O14	0.91	2.45	3.008 (2)	120
O32—H32⋯N25	0.93	1.67	2.595 (2)	176
C1—H1⋯O33	1.00	2.57	3.249 (3)	125
C5—H5⋯O32^i^	0.95	2.58	3.442 (2)	152
C16—H16⋯O32^ii^	0.95	2.47	3.340 (2)	153

Symmetry codes: (i) 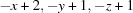; (ii) 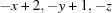.

**Table 2:** Experimental details

Crystal data	
Chemical formula	C_26_H_32_N_2_O_4_·C_2_H_4_O_2_
*M*_r_	496.59
Crystal system, space group	Triclinic, *P* 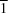
Temperature (K)	120
*a*, *b*, *c* (Å)	9.4610 (8), 11.673 (1), 12.9862 (11)
α, β, γ (°)	83.780 (2), 79.998 (2), 67.335 (2)
*V* (Å^3^)	1301.95 (19)
*Z*	2
Radiation type	Mo *K*α
μ (mm^−1^)	0.09
Crystal size (mm)	0.30 × 0.20 × 0.20

Data collection	
Diffractometer	Bruker APEXII CCD
Absorption correction	Multi-scan (*SADABS*; Sheldrick, 2003 ▸)
*T*_min_, *T*_max_	0.946, 0.963
No. of measured, independent and observed [*I* > 2σ(*I*)] reflections	17314, 7954, 5223
*R*_int_	0.046
(sin θ/λ)_max_ (Å^−1^)	0.716

Refinement	
*R*[*F* ^2^ > 2σ(*F* ^2^)], *wR*(*F* ^2^), *S*	0.065, 0.143, 1.01
No. of reflections	7954
No. of parameters	327
H-atom treatment	H-atom parameters constrained
Δρ_max_, Δρ_min_ (e Å^−3^)	0.39, −0.29

Computer programs: *APEX2* (Bruker, 2005 ▸), *SAINT* (Bruker, 2001 ▸), *SHELXTL* (Sheldrick, 2008 ▸).

## supplementary crystallographic information

### Crystal data

**Table:**

C_26_H_32_N_2_O_4_·C_2_H_4_O_2_	*Z* = 2
*M**_r_* = 496.59	*F*(000) = 532
Triclinic, *P*1	*D*_x_ = 1.267 Mg m^−^^3^
*a* = 9.4610 (8) Å	Mo *K*α radiation, λ = 0.71073 Å
*b* = 11.673 (1) Å	Cell parameters from 3303 reflections
*c* = 12.9862 (11) Å	θ = 2.4–29.1°
α = 83.780 (2)°	µ = 0.09 mm^−^^1^
β = 79.998 (2)°	*T* = 120 K
γ = 67.335 (2)°	Prism, colourless
*V* = 1301.95 (19) Å^3^	0.30 × 0.20 × 0.20 mm

### Data collection

**Table:**

Bruker APEXII CCD diffractometer	5223 reflections with *I* > 2σ(*I*)
Radiation source: fine-focus sealed tube	*R*_int_ = 0.046
φ and ω scans	θ_max_ = 30.6°, θ_min_ = 1.6°
Absorption correction: multi-scan (*SADABS*; Sheldrick, 2003)	*h* = −13→13
*T*_min_ = 0.946, *T*_max_ = 0.963	*k* = −16→16
17314 measured reflections	*l* = −18→18
7954 independent reflections	

### Refinement

**Table:**

Refinement on *F*^2^	Primary atom site location: difference Fourier map
Least-squares matrix: full	Secondary atom site location: difference Fourier map
*R*[*F*^2^ > 2σ(*F*^2^)] = 0.065	Hydrogen site location: mixed
*wR*(*F*^2^) = 0.143	H-atom parameters constrained
*S* = 1.01	*w* = 1/[σ^2^(*F*_o_^2^) + (0.0492*P*)^2^ + 0.4449*P*] where *P* = (*F*_o_^2^ + 2*F*_c_^2^)/3
7954 reflections	(Δ/σ)_max_ = 0.001
327 parameters	Δρ_max_ = 0.39 e Å^−^^3^
0 restraints	Δρ_min_ = −0.29 e Å^−^^3^

### Special details

**Table:**

Geometry. All esds (except the esd in the dihedral angle between two l.s. planes) are estimated using the full covariance matrix. The cell esds are taken into account individually in the estimation of esds in distances, angles and torsion angles; correlations between esds in cell parameters are only used when they are defined by crystal symmetry. An approximate (isotropic) treatment of cell esds is used for estimating esds involving l.s. planes.

### Fractional atomic coordinates and isotropic or equivalent isotropic displacement parameters (Å^2^)

**Table:**

	*x*	*y*	*z*	*U*_iso_*/*U*_eq_	
C1	0.7020 (2)	0.80143 (16)	0.32331 (13)	0.0151 (3)	
H1	0.5943	0.8030	0.3432	0.018*	
C2	0.7882 (2)	0.73774 (16)	0.41389 (13)	0.0156 (3)	
C3	0.7109 (2)	0.70361 (17)	0.50551 (14)	0.0187 (4)	
H3	0.6039	0.7188	0.5099	0.022*	
C4	0.7870 (2)	0.64764 (17)	0.59081 (14)	0.0219 (4)	
H4	0.7321	0.6262	0.6534	0.026*	
C5	0.9433 (2)	0.62344 (17)	0.58399 (14)	0.0217 (4)	
H5	0.9957	0.5846	0.6421	0.026*	
C6	1.0246 (2)	0.65524 (16)	0.49323 (14)	0.0199 (4)	
H6	1.1322	0.6374	0.4887	0.024*	
C7	0.9465 (2)	0.71355 (16)	0.40895 (14)	0.0171 (3)	
O8	1.01351 (14)	0.75192 (12)	0.31660 (10)	0.0216 (3)	
C9	1.1750 (2)	0.73063 (18)	0.30576 (15)	0.0212 (4)	
H9A	1.2378	0.6404	0.3071	0.025*	
H9B	1.1961	0.7677	0.3636	0.025*	
C10	1.2134 (2)	0.79095 (18)	0.20263 (15)	0.0236 (4)	
H10A	1.1391	0.8779	0.1975	0.028*	
H10B	1.3188	0.7918	0.1963	0.028*	
O11	1.20538 (16)	0.72247 (12)	0.12144 (10)	0.0241 (3)	
C12	1.1807 (2)	0.79105 (18)	0.02482 (15)	0.0244 (4)	
H12A	1.2802	0.7912	−0.0144	0.029*	
H12B	1.1104	0.8782	0.0374	0.029*	
C13	1.1102 (2)	0.73105 (19)	−0.03698 (15)	0.0240 (4)	
H13A	1.1011	0.7713	−0.1079	0.029*	
H13B	1.1748	0.6416	−0.0437	0.029*	
O14	0.95979 (15)	0.74700 (12)	0.01989 (10)	0.0232 (3)	
C15	0.8744 (2)	0.69058 (16)	−0.01385 (14)	0.0194 (4)	
C16	0.9239 (2)	0.61671 (17)	−0.10028 (14)	0.0226 (4)	
H16	1.0216	0.6039	−0.1414	0.027*	
C17	0.8277 (2)	0.56197 (18)	−0.12541 (15)	0.0258 (4)	
H17	0.8612	0.5108	−0.1838	0.031*	
C18	0.6844 (2)	0.58076 (18)	−0.06688 (15)	0.0256 (4)	
H18	0.6194	0.5435	−0.0851	0.031*	
C19	0.6368 (2)	0.65503 (17)	0.01915 (14)	0.0215 (4)	
H19	0.5384	0.6682	0.0595	0.026*	
C20	0.7302 (2)	0.71058 (16)	0.04736 (13)	0.0166 (3)	
C21	0.6760 (2)	0.78938 (16)	0.14221 (13)	0.0152 (3)	
H21	0.5681	0.7948	0.1691	0.018*	
C22	0.6672 (2)	0.92585 (16)	0.11686 (14)	0.0166 (3)	
H22	0.7129	0.9357	0.0426	0.020*	
C23	0.7502 (2)	0.96020 (16)	0.19043 (14)	0.0173 (4)	
O23	0.84543 (15)	1.00757 (12)	0.16251 (10)	0.0237 (3)	
C24	0.6875 (2)	0.93985 (16)	0.30267 (14)	0.0166 (3)	
H24	0.7460	0.9601	0.3505	0.020*	
N25	0.76982 (17)	0.72799 (13)	0.22760 (11)	0.0149 (3)	
H25	0.8675	0.7259	0.2065	0.018*	
C26	0.4975 (2)	1.01694 (17)	0.13699 (14)	0.0191 (4)	
H26A	0.4925	1.1032	0.1216	0.023*	
H26B	0.4366	1.0002	0.0898	0.023*	
N27	0.43102 (17)	1.00357 (14)	0.24556 (11)	0.0173 (3)	
C28	0.5164 (2)	1.02988 (17)	0.31802 (14)	0.0187 (4)	
H28A	0.4688	1.0206	0.3910	0.022*	
H28B	0.5107	1.1166	0.3054	0.022*	
C29	0.2644 (2)	1.07837 (17)	0.26161 (15)	0.0208 (4)	
H29A	0.2169	1.0627	0.2052	0.025*	
H29B	0.2495	1.1674	0.2557	0.025*	
C30	0.1803 (2)	1.05181 (18)	0.36677 (16)	0.0244 (4)	
H30A	0.2005	1.0930	0.4222	0.029*	
H30B	0.2199	0.9612	0.3837	0.029*	
C31	0.0058 (2)	1.0991 (2)	0.36409 (18)	0.0352 (5)	
H31A	−0.0476	1.0901	0.4344	0.053*	
H31B	−0.0152	1.0506	0.3158	0.053*	
H31C	−0.0314	1.1869	0.3404	0.053*	
O32	0.75677 (15)	0.50970 (13)	0.27083 (11)	0.0263 (3)	
H32	0.7652	0.5868	0.2569	0.039*	
O33	0.51282 (18)	0.61820 (16)	0.33797 (16)	0.0489 (5)	
C32	0.6186 (2)	0.5196 (2)	0.31786 (19)	0.0335 (5)	
C33	0.6036 (3)	0.3964 (3)	0.3473 (3)	0.0692 (10)	
H33A	0.5385	0.4009	0.4154	0.104*	
H33B	0.7064	0.3320	0.3516	0.104*	
H33C	0.5561	0.3759	0.2942	0.104*	

### Atomic displacement parameters (Å^2^)

**Table:**

	*U*^11^	*U*^22^	*U*^33^	*U*^12^	*U*^13^	*U*^23^
C1	0.0143 (8)	0.0166 (8)	0.0132 (8)	−0.0045 (7)	−0.0020 (6)	−0.0009 (6)
C2	0.0168 (8)	0.0149 (8)	0.0142 (8)	−0.0040 (7)	−0.0046 (7)	−0.0011 (6)
C3	0.0187 (9)	0.0212 (9)	0.0175 (9)	−0.0094 (7)	−0.0024 (7)	0.0006 (7)
C4	0.0291 (10)	0.0221 (9)	0.0147 (8)	−0.0102 (8)	−0.0041 (7)	0.0025 (7)
C5	0.0282 (10)	0.0178 (9)	0.0189 (9)	−0.0050 (8)	−0.0123 (8)	0.0015 (7)
C6	0.0181 (9)	0.0183 (9)	0.0222 (9)	−0.0030 (7)	−0.0077 (7)	−0.0024 (7)
C7	0.0181 (9)	0.0166 (8)	0.0161 (8)	−0.0052 (7)	−0.0031 (7)	−0.0023 (7)
O8	0.0138 (6)	0.0316 (7)	0.0192 (6)	−0.0089 (6)	−0.0033 (5)	0.0025 (6)
C9	0.0120 (8)	0.0273 (10)	0.0250 (10)	−0.0068 (7)	−0.0031 (7)	−0.0051 (8)
C10	0.0166 (9)	0.0253 (10)	0.0305 (10)	−0.0103 (8)	−0.0002 (8)	−0.0037 (8)
O11	0.0293 (8)	0.0220 (7)	0.0208 (7)	−0.0099 (6)	−0.0040 (6)	0.0011 (6)
C12	0.0186 (9)	0.0263 (10)	0.0253 (10)	−0.0085 (8)	0.0004 (8)	0.0062 (8)
C13	0.0179 (9)	0.0302 (10)	0.0186 (9)	−0.0068 (8)	0.0044 (7)	0.0009 (8)
O14	0.0183 (7)	0.0290 (7)	0.0226 (7)	−0.0103 (6)	0.0037 (5)	−0.0076 (6)
C15	0.0238 (10)	0.0161 (8)	0.0164 (8)	−0.0053 (7)	−0.0042 (7)	0.0001 (7)
C16	0.0274 (10)	0.0201 (9)	0.0139 (8)	−0.0031 (8)	−0.0008 (7)	0.0002 (7)
C17	0.0395 (12)	0.0206 (9)	0.0148 (9)	−0.0068 (9)	−0.0072 (8)	−0.0015 (7)
C18	0.0344 (11)	0.0219 (9)	0.0226 (10)	−0.0101 (9)	−0.0092 (9)	−0.0025 (8)
C19	0.0242 (10)	0.0210 (9)	0.0205 (9)	−0.0085 (8)	−0.0068 (8)	0.0006 (7)
C20	0.0197 (9)	0.0171 (8)	0.0123 (8)	−0.0048 (7)	−0.0055 (7)	0.0006 (7)
C21	0.0131 (8)	0.0183 (8)	0.0143 (8)	−0.0057 (7)	−0.0032 (6)	0.0002 (7)
C22	0.0170 (8)	0.0159 (8)	0.0153 (8)	−0.0040 (7)	−0.0041 (7)	0.0010 (7)
C23	0.0156 (8)	0.0121 (8)	0.0215 (9)	−0.0020 (7)	−0.0043 (7)	0.0009 (7)
O23	0.0236 (7)	0.0240 (7)	0.0266 (7)	−0.0129 (6)	−0.0044 (6)	0.0033 (6)
C24	0.0152 (8)	0.0168 (8)	0.0172 (8)	−0.0041 (7)	−0.0043 (7)	−0.0025 (7)
N25	0.0134 (7)	0.0183 (7)	0.0119 (7)	−0.0047 (6)	−0.0023 (5)	0.0004 (6)
C26	0.0189 (9)	0.0189 (9)	0.0169 (9)	−0.0035 (7)	−0.0054 (7)	0.0006 (7)
N27	0.0144 (7)	0.0186 (7)	0.0160 (7)	−0.0021 (6)	−0.0035 (6)	−0.0021 (6)
C28	0.0181 (9)	0.0171 (8)	0.0185 (9)	−0.0031 (7)	−0.0040 (7)	−0.0026 (7)
C29	0.0160 (9)	0.0187 (9)	0.0242 (9)	−0.0018 (7)	−0.0035 (7)	−0.0037 (7)
C30	0.0191 (9)	0.0236 (10)	0.0287 (10)	−0.0069 (8)	0.0011 (8)	−0.0055 (8)
C31	0.0198 (10)	0.0460 (13)	0.0418 (13)	−0.0136 (10)	0.0039 (9)	−0.0197 (11)
O32	0.0226 (7)	0.0234 (7)	0.0322 (8)	−0.0096 (6)	−0.0017 (6)	0.0025 (6)
O33	0.0194 (8)	0.0413 (10)	0.0799 (14)	−0.0085 (8)	−0.0020 (8)	0.0035 (9)
C32	0.0218 (10)	0.0343 (12)	0.0450 (13)	−0.0121 (10)	−0.0105 (10)	0.0106 (10)
C33	0.0372 (15)	0.0426 (15)	0.129 (3)	−0.0252 (13)	−0.0097 (17)	0.0252 (18)

### Geometric parameters (Å, º)

**Table:**

C1—N25	1.490 (2)	C19—C20	1.394 (2)
C1—C2	1.510 (2)	C19—H19	0.9500
C1—C24	1.565 (2)	C20—C21	1.513 (2)
C1—H1	1.0000	C21—N25	1.487 (2)
C2—C3	1.389 (2)	C21—C22	1.564 (2)
C2—C7	1.404 (2)	C21—H21	1.0000
C3—C4	1.389 (2)	C22—C23	1.508 (2)
C3—H3	0.9500	C22—C26	1.542 (2)
C4—C5	1.383 (3)	C22—H22	1.0000
C4—H4	0.9500	C23—O23	1.215 (2)
C5—C6	1.389 (3)	C23—C24	1.505 (2)
C5—H5	0.9500	C24—C28	1.545 (2)
C6—C7	1.392 (2)	C24—H24	1.0000
C6—H6	0.9500	N25—H25	0.9090
C7—O8	1.371 (2)	C26—N27	1.459 (2)
O8—C9	1.434 (2)	C26—H26A	0.9900
C9—C10	1.499 (3)	C26—H26B	0.9900
C9—H9A	0.9900	N27—C28	1.466 (2)
C9—H9B	0.9900	N27—C29	1.468 (2)
C10—O11	1.419 (2)	C28—H28A	0.9900
C10—H10A	0.9900	C28—H28B	0.9900
C10—H10B	0.9900	C29—C30	1.522 (3)
O11—C12	1.418 (2)	C29—H29A	0.9900
C12—C13	1.499 (3)	C29—H29B	0.9900
C12—H12A	0.9900	C30—C31	1.531 (3)
C12—H12B	0.9900	C30—H30A	0.9900
C13—O14	1.437 (2)	C30—H30B	0.9900
C13—H13A	0.9900	C31—H31A	0.9800
C13—H13B	0.9900	C31—H31B	0.9800
O14—C15	1.369 (2)	C31—H31C	0.9800
C15—C16	1.392 (3)	O32—C32	1.310 (2)
C15—C20	1.402 (3)	O32—H32	0.9300
C16—C17	1.392 (3)	O33—C32	1.217 (3)
C16—H16	0.9500	C32—C33	1.502 (3)
C17—C18	1.383 (3)	C33—H33A	0.9800
C17—H17	0.9500	C33—H33B	0.9800
C18—C19	1.392 (3)	C33—H33C	0.9800
C18—H18	0.9500		
			
N25—C1—C2	111.06 (14)	C15—C20—C21	121.60 (15)
N25—C1—C24	112.37 (13)	N25—C21—C20	111.21 (14)
C2—C1—C24	112.90 (14)	N25—C21—C22	111.78 (13)
N25—C1—H1	106.7	C20—C21—C22	113.66 (14)
C2—C1—H1	106.7	N25—C21—H21	106.6
C24—C1—H1	106.7	C20—C21—H21	106.6
C3—C2—C7	118.35 (16)	C22—C21—H21	106.6
C3—C2—C1	120.22 (16)	C23—C22—C26	105.47 (14)
C7—C2—C1	121.42 (15)	C23—C22—C21	110.33 (14)
C2—C3—C4	121.25 (17)	C26—C22—C21	109.81 (14)
C2—C3—H3	119.4	C23—C22—H22	110.4
C4—C3—H3	119.4	C26—C22—H22	110.4
C5—C4—C3	119.51 (17)	C21—C22—H22	110.4
C5—C4—H4	120.2	O23—C23—C24	124.69 (16)
C3—C4—H4	120.2	O23—C23—C22	124.22 (17)
C4—C5—C6	120.77 (16)	C24—C23—C22	110.82 (15)
C4—C5—H5	119.6	C23—C24—C28	105.96 (14)
C6—C5—H5	119.6	C23—C24—C1	109.72 (14)
C5—C6—C7	119.24 (17)	C28—C24—C1	111.30 (14)
C5—C6—H6	120.4	C23—C24—H24	109.9
C7—C6—H6	120.4	C28—C24—H24	109.9
O8—C7—C6	124.44 (16)	C1—C24—H24	109.9
O8—C7—C2	114.69 (15)	C21—N25—C1	109.49 (13)
C6—C7—C2	120.87 (16)	C21—N25—H25	108.3
C7—O8—C9	117.94 (14)	C1—N25—H25	109.1
O8—C9—C10	106.49 (14)	N27—C26—C22	110.50 (14)
O8—C9—H9A	110.4	N27—C26—H26A	109.6
C10—C9—H9A	110.4	C22—C26—H26A	109.6
O8—C9—H9B	110.4	N27—C26—H26B	109.6
C10—C9—H9B	110.4	C22—C26—H26B	109.6
H9A—C9—H9B	108.6	H26A—C26—H26B	108.1
O11—C10—C9	108.52 (15)	C26—N27—C28	111.21 (14)
O11—C10—H10A	110.0	C26—N27—C29	110.59 (14)
C9—C10—H10A	110.0	C28—N27—C29	113.43 (14)
O11—C10—H10B	110.0	N27—C28—C24	110.26 (14)
C9—C10—H10B	110.0	N27—C28—H28A	109.6
H10A—C10—H10B	108.4	C24—C28—H28A	109.6
C12—O11—C10	114.24 (15)	N27—C28—H28B	109.6
O11—C12—C13	108.19 (15)	C24—C28—H28B	109.6
O11—C12—H12A	110.1	H28A—C28—H28B	108.1
C13—C12—H12A	110.1	N27—C29—C30	114.01 (15)
O11—C12—H12B	110.1	N27—C29—H29A	108.7
C13—C12—H12B	110.1	C30—C29—H29A	108.7
H12A—C12—H12B	108.4	N27—C29—H29B	108.7
O14—C13—C12	106.24 (15)	C30—C29—H29B	108.7
O14—C13—H13A	110.5	H29A—C29—H29B	107.6
C12—C13—H13A	110.5	C29—C30—C31	110.60 (17)
O14—C13—H13B	110.5	C29—C30—H30A	109.5
C12—C13—H13B	110.5	C31—C30—H30A	109.5
H13A—C13—H13B	108.7	C29—C30—H30B	109.5
C15—O14—C13	118.86 (14)	C31—C30—H30B	109.5
O14—C15—C16	124.32 (17)	H30A—C30—H30B	108.1
O14—C15—C20	114.57 (15)	C30—C31—H31A	109.5
C16—C15—C20	121.10 (17)	C30—C31—H31B	109.5
C15—C16—C17	118.93 (18)	H31A—C31—H31B	109.5
C15—C16—H16	120.5	C30—C31—H31C	109.5
C17—C16—H16	120.5	H31A—C31—H31C	109.5
C18—C17—C16	121.24 (18)	H31B—C31—H31C	109.5
C18—C17—H17	119.4	C32—O32—H32	111.9
C16—C17—H17	119.4	O33—C32—O32	124.0 (2)
C17—C18—C19	119.05 (19)	O33—C32—C33	122.7 (2)
C17—C18—H18	120.5	O32—C32—C33	113.3 (2)
C19—C18—H18	120.5	C32—C33—H33A	109.5
C18—C19—C20	121.43 (18)	C32—C33—H33B	109.5
C18—C19—H19	119.3	H33A—C33—H33B	109.5
C20—C19—H19	119.3	C32—C33—H33C	109.5
C19—C20—C15	118.25 (16)	H33A—C33—H33C	109.5
C19—C20—C21	120.15 (16)	H33B—C33—H33C	109.5
			
N25—C1—C2—C3	−120.84 (17)	C19—C20—C21—N25	111.18 (17)
C24—C1—C2—C3	111.90 (18)	C15—C20—C21—N25	−68.4 (2)
N25—C1—C2—C7	60.4 (2)	C19—C20—C21—C22	−121.62 (17)
C24—C1—C2—C7	−66.9 (2)	C15—C20—C21—C22	58.8 (2)
C7—C2—C3—C4	0.4 (3)	N25—C21—C22—C23	−2.93 (19)
C1—C2—C3—C4	−178.42 (17)	C20—C21—C22—C23	−129.83 (15)
C2—C3—C4—C5	−1.1 (3)	N25—C21—C22—C26	−118.76 (15)
C3—C4—C5—C6	0.5 (3)	C20—C21—C22—C26	114.33 (16)
C4—C5—C6—C7	0.8 (3)	C26—C22—C23—O23	−110.80 (19)
C5—C6—C7—O8	178.32 (17)	C21—C22—C23—O23	130.67 (18)
C5—C6—C7—C2	−1.5 (3)	C26—C22—C23—C24	63.50 (17)
C3—C2—C7—O8	−178.95 (15)	C21—C22—C23—C24	−55.02 (18)
C1—C2—C7—O8	−0.1 (2)	O23—C23—C24—C28	111.02 (19)
C3—C2—C7—C6	0.9 (3)	C22—C23—C24—C28	−63.25 (17)
C1—C2—C7—C6	179.71 (16)	O23—C23—C24—C1	−128.72 (18)
C6—C7—O8—C9	0.1 (2)	C22—C23—C24—C1	57.00 (18)
C2—C7—O8—C9	179.88 (15)	N25—C1—C24—C23	−1.16 (19)
C7—O8—C9—C10	−175.18 (15)	C2—C1—C24—C23	125.40 (16)
O8—C9—C10—O11	−69.33 (18)	N25—C1—C24—C28	115.80 (15)
C9—C10—O11—C12	157.26 (15)	C2—C1—C24—C28	−117.65 (16)
C10—O11—C12—C13	−156.70 (15)	C20—C21—N25—C1	−173.72 (14)
O11—C12—C13—O14	65.88 (19)	C22—C21—N25—C1	58.06 (17)
C12—C13—O14—C15	−173.95 (15)	C2—C1—N25—C21	176.47 (13)
C13—O14—C15—C16	0.5 (3)	C24—C1—N25—C21	−55.99 (17)
C13—O14—C15—C20	179.35 (15)	C23—C22—C26—N27	−60.27 (18)
O14—C15—C16—C17	178.54 (17)	C21—C22—C26—N27	58.60 (18)
C20—C15—C16—C17	−0.2 (3)	C22—C26—N27—C28	60.27 (18)
C15—C16—C17—C18	0.6 (3)	C22—C26—N27—C29	−172.75 (14)
C16—C17—C18—C19	−0.5 (3)	C26—N27—C28—C24	−59.54 (18)
C17—C18—C19—C20	0.0 (3)	C29—N27—C28—C24	175.04 (14)
C18—C19—C20—C15	0.4 (3)	C23—C24—C28—N27	59.27 (18)
C18—C19—C20—C21	−179.16 (17)	C1—C24—C28—N27	−59.95 (18)
O14—C15—C20—C19	−179.19 (15)	C26—N27—C29—C30	169.32 (15)
C16—C15—C20—C19	−0.3 (3)	C28—N27—C29—C30	−64.9 (2)
O14—C15—C20—C21	0.4 (2)	N27—C29—C30—C31	−161.57 (16)
C16—C15—C20—C21	179.25 (16)		

### Hydrogen-bond geometry (Å, º)

**Table:**

*D*—H···*A*	*D*—H	H···*A*	*D*···*A*	*D*—H···*A*
N25—H25···O8	0.91	2.27	2.867 (2)	123
N25—H25···O14	0.91	2.45	3.008 (2)	120
O32—H32···N25	0.93	1.67	2.595 (2)	176
C1—H1···O33	1.00	2.57	3.249 (3)	125
C5—H5···O32^i^	0.95	2.58	3.442 (2)	152
C16—H16···O32^ii^	0.95	2.47	3.340 (2)	153

Symmetry codes: (i) −*x*+2, −*y*+1, −*z*+1; (ii) −*x*+2, −*y*+1, −*z*.
